# Supplementary material for: Phylogeography and Population Demography of Parrotia subaequalis, a Hamamelidaceous Tertiary Relict ‘Living Fossil’ Tree Endemic to East Asia Refugia: Implications from Molecular Data and Ecological Niche Modeling
Source: Plants (Basel). 2025 Jun 7;14(12):1754. doi: 10.3390/plants14121754 (PMC12197062; doi:10.3390/plants14121754)
Supplement: Supplementary file 1 [file plants-14-01754-s001.zip › Supplementary Figure Legends.pdf]

Supplementary Figure Legends

Figure S1. The correlation among genetic diversity (*h* and *π*) of *Parrotia subaequalis* and the latitudinal/longitudinal coordinates on the level of cpDNA.

Figure S2. Principal coordinate analysis (PCoA) based on pairwise genetic distance estimates for all populations of *Parrotia subaequali*s.

Figure S3. Mismatch distribution of all populations of *Parrotia subaequalis.*

Figure S4. The response curve of temperature seasonality (BIO4).

Figure S5. The response curve of max temperature of warmest month (BIO5).

Figure S6. The response curve of precipitation of driest quarter (BIO17).

Figure S7. The response curve of precipitation of warmest quarter (BIO18).
