# Supplementary material for: Phylogeography and Population Demography of Parrotia subaequalis, a Hamamelidaceous Tertiary Relict ‘Living Fossil’ Tree Endemic to East Asia Refugia: Implications from Molecular Data and Ecological Niche Modeling
Source: Plants (Basel). 2025 Jun 7;14(12):1754. doi: 10.3390/plants14121754 (PMC12197062; doi:10.3390/plants14121754)
Supplement: Supplementary file 1 [file plants-14-01754-s001.zip › Supplementary Table Legends.pdf]

Supplementary Table Legends

Table S1. The sampling information of *Parrotia subaequalis* populations in this study and the number of individuals used for cpDNA and EST-SSR analyses.

Table S2. The information of cpDNA primers selected for screening in this study.

Table S3. Information of the 16 polymorphic EST-SSR markers used in this study.

Table S4. 19 bioclimatic variables for ENM analysis.

Table S5. Chloroplast DNA sequence polymorphisms detected in *Parrotia subaequalis* at three intergenic spacer (*psbC–psbZ*, *accD–psaI*, *ndhD–psaC*) regions, identifying 13 chlorotypes (H1–H13).

Table S6. Genetic characteristics of cpDNA for 21 *Parrotia subaequalis* populations.

Table S7. Analysis of molecular variance (AMOVA) of cpDNA sequences (*psbC–psbZ*, *accD–psaI*, *ndhD–psaC*) data of the populations of *Parrotia subaequalis*.

Table S8. Summary statistics of the genetic diversity of the 16 polymorphic EST-SSR loci.

Table S9. Summary statistics of the genetic diversity of the 21 populations of *Parrotia subaequalis* based on the 16 polymorphic EST-SSR loci.

Table S10. Analysis of molecular variance (AMOVA) of the populations of *Parrotia subaequalis* based on 16 EST-SSR loci.

Table S11. Detection of the bottleneck effect of the populations of *Parrotia subaequalis* based on the Infinite allele model (IAM), Stepwise mutation model (SMM), Two-phased model of mutation (TPM) and Mode-Shift method.
