# Supplementary material for: Phylogeography and Population Demography of Parrotia subaequalis, a Hamamelidaceous Tertiary Relict ‘Living Fossil’ Tree Endemic to East Asia Refugia: Implications from Molecular Data and Ecological Niche Modeling
Source: Plants (Basel). 2025 Jun 7;14(12):1754. doi: 10.3390/plants14121754 (PMC12197062; doi:10.3390/plants14121754)
Supplement: Supplementary file 1 [file plants-14-01754-s001.zip › Table S1.pdf]

Table S1. The sampling information of *Parrotia subaequalis* populations in this study and the number of individuals used for cpDNA and EST-SSR analyses.

Population ID number	Population code	Sampling locations	Latitude (N)	Longtitude (E)	Altitude (m)	Voucher number	cpDNA/EST-SSR
1	SYC	Shangyang Village, Zhejiang Province, China	29°50'06"	121°58'12"	300-450	Z200510	24/22
2	TXC	Tangxi Village, Zhejiang Province, China	29°21'22"	121°28'38"	549	Z200512	6/6
3	SLG	Mountain Shangliang, Zhejiang Province, China	29°42'45"	121°12'04"	823-926	Z190616	18/16
4	DLX	Mountain Dalong, Jiangsu Province, China	31°14'46"	119°44'39"	211-300	Z180518	7/20
5	SJD	Shangjuan Cave, Jiangsu Province, China	31°17'55"	119°40'03"	32	Z180519	20/20
6	GDS	Mountain Guodi, Jiangsu Province, China	31°09'26"	119°30'36"	493-524	Z190423	20/20
7	LWS	Mountain Longwang, Zhejiang Province, China	30°23'44"	119°24'46"	658-1073	Z180520	17/26
8	QSW	Mountain Qishuwan, Zhejiang Province, China	30°09'01"	118°51'42"	689-876	Z190505	12/20
9	ZXC	Zhuxian Village, Anhui Province, China	30°12'33"	118°54'09"	718	Z190506	16/18
10	SJW	Mountain Shuijingwan, Anhui Province, China	30°10'50"	118°51'53"	677-769	Z190507	8/19
11	LHJ	Lianhua Ravine, Anhui Province, China	31°14'35"	116°52'18"	402-539	Z200611	16/20
12	QL	Mountain Qiling, Anhui Province, China	31°11'53"	116°56'55"	361-485	Z200612	19/22
13	WFS	Mountain Wanfo, Anhui Province, China	31°04'31"	116°33'53"	452-610	Z180522	19/20
14	YSH	Yingshan River, Anhui Province, China	30°59'42"	116°21'37"	440	Z190514	17/20
15	TTS	Mountain Tuantu, Anhui Province, China	31°02'35"	116°23'15"	751-805	Z190515	16/20
16	TJZ	Tuanjie Village, Anhui Province, China	31°04'16"	116°17'57"	400-580	Z190516	17/20
17	TX	Mountain Tianxia, Anhui Province, China	30°49'19"	116°03'27"	372-404	Z190517	11/20
18	DXG	Daxiagu Village, Anhui Province, China	31°15'31"	115°54'54"	405-485	Z190518	16/17
19	JCY	Mountain Jiucai, Anhui Province, China	31°12'50"	115°54'11"	629-769	Z190519	24/26
20	HNZ	Mountain Huaniang, Anhui Province, China	31°25'20"	115°29'45"	586-617	Z190520	14/16
21	WLS	Wulishan Village, Henan Province, China	31°27'23"	115°16'24"	199-304	Z200608	22/22
