# Supplementary material for: Phylogeography and Population Demography of Parrotia subaequalis, a Hamamelidaceous Tertiary Relict ‘Living Fossil’ Tree Endemic to East Asia Refugia: Implications from Molecular Data and Ecological Niche Modeling
Source: Plants (Basel). 2025 Jun 7;14(12):1754. doi: 10.3390/plants14121754 (PMC12197062; doi:10.3390/plants14121754)
Supplement: Supplementary file 1 [file plants-14-01754-s001.zip › Table S10.pdf]

Table S10. Analysis of molecular variance (AMOVA) of the populations of *Parrotia subaequalis* based on 16 EST-SSR loci.

Source of variation	d.f.	Sum of squares	Variance components	Percentage of variation (%)
Among groups	2	80.568	0.13837	3.98
Among populations within group	20	1391.733	0.08109	2.33
Within populations	409	1335.500	3.25732	93.69
Total	431	2807.801	3.47677	100
