# Supplementary material for: Phylogeography and Population Demography of Parrotia subaequalis, a Hamamelidaceous Tertiary Relict ‘Living Fossil’ Tree Endemic to East Asia Refugia: Implications from Molecular Data and Ecological Niche Modeling
Source: Plants (Basel). 2025 Jun 7;14(12):1754. doi: 10.3390/plants14121754 (PMC12197062; doi:10.3390/plants14121754)
Supplement: Supplementary file 1 [file plants-14-01754-s001.zip › Table S11.pdf]

Table S11. Detection of the bottleneck effect of the populations of *Parrotia subaequalis* based on the Infinite allele model (IAM), Stepwise mutation model (SMM), Two-phased model of mutation (TPM) and Mode-Shift method.

Population code	IAM	SMM	TPM	Mode-Shift method
SYC	0.277	0.561	0.847	L-shaped
TXC	0.034*	0.519	0.064	shifted
SLG	0.015*	0.208	0.041*	L-shaped
DLX	0.277	0.561	0.978	L-shaped
SJD	0.127	0.685	0.414	L-shaped
GDS	0.068	0.455	0.305	L-shaped
LWS	0.211	0.528	0.782	L-shaped
QSW	0.040*	0.414	0.127	L-shaped
ZXC	0.002**	0.095	0.048*	shifted
SJW	0.358	0.463	0.855	L-shaped
LHJ	0.000***	0.003**	0.002**	shifted
QL	0.001***	0.229	0.008**	L-shaped
WFS	0.277	0.679	0.639	L-shaped
YSH	0.130	0.274	0.980	L-shaped
TTS	0.002**	0.241	0.025*	L-shaped
TJZ	0.298	0.669	0.821	L-shaped
TX	0.241	0.670	0.715	L-shaped
DXG	0.121	0.679	0.524	L-shaped
JCY	0.034*	0.597	0.231	L-shaped
HNZ	0.744	0.083	0.464	L-shaped
WLS	0.119	0.670	0.296	L-shaped

Note: $^{*}P < 0.05$; $^{**}P < 0.01$; $^{***}P < 0.001$.
