# Supplementary material for: Phylogeography and Population Demography of Parrotia subaequalis, a Hamamelidaceous Tertiary Relict ‘Living Fossil’ Tree Endemic to East Asia Refugia: Implications from Molecular Data and Ecological Niche Modeling
Source: Plants (Basel). 2025 Jun 7;14(12):1754. doi: 10.3390/plants14121754 (PMC12197062; doi:10.3390/plants14121754)
Supplement: Supplementary file 1 [file plants-14-01754-s001.zip › Table S2.pdf]

Table S2. The information of cpDNA primers selected for screening in this study.

Primer	Primer Sequences (5'–3')	Optimal T_m
*psb*C–*psb*Z	Forward: CTATCAACCACTCGGCCATCT<br>Reverse: ATCCTCCAAGCTACCAACAAAA	56°C
*acc*D–*psa*I	Forward: TAGGCAAGGCAACTAAAA<br>Reverse: CTAAAGGCACCAAAACAG	55°C
*ndh*D–*psa*C	Forward: AATCGGTGATTTGACAGC<br>Reverse: ATACCTTGGGACGGATGT	56°C
