# Supplementary material for: Phylogeography and Population Demography of Parrotia subaequalis, a Hamamelidaceous Tertiary Relict ‘Living Fossil’ Tree Endemic to East Asia Refugia: Implications from Molecular Data and Ecological Niche Modeling
Source: Plants (Basel). 2025 Jun 7;14(12):1754. doi: 10.3390/plants14121754 (PMC12197062; doi:10.3390/plants14121754)
Supplement: Supplementary file 1 [file plants-14-01754-s001.zip › Table S3.pdf]

Table S3. Information of the 16 polymorphic EST-SSR markers used in this study.

Locus	Primer sequences (5'- 3')	Repeat motif	Product size (bp)	Optimal T_m (°C)	Fluorescent dye*	GenBank accession no.
PasE6	F: GCCAAACAACACCAACAAACC	$(AAG)_5$	153	53	FAM	MK238352
	R: GTCGCCGATGGAGGTAAGAC					
PasE27	F: TCTCTTCACCCATCTCCCCAT	$(AC)_7$	178	51	HEX	MK238354
	R: GTTGGGTGGGTTTCAGAGCT					
PasE83	F: TGGCAGACAACGAAGGATGG	$(AGC)_5$	167	52	HEX	MK238355
	R: CCATCTCGGTTGCCACTTCT					
PasE108	F: CTCCGTTGACCAAAACTGGAC	$(AT)_6$	208	59	TAMRA	MK238356
	R: CCAAAGAATCCTGCAAAGAAAGC					
PasE156	F: GCCGATCAAGATGCGGTTTC	$(ATA)_8$	202	59	TAMRA	MK238357
	R: CGGGGCTCTTCTTCTCCATG					
PasE178	F: CTAGTCCCAGCCAAACAGCA	$(CAC)_6$	129	53	ROX	MK238359
	R: CATCGAGTGGCTCCAGAGTG					
PasE188	F: GACCCTGCCCATCTTCTGTC	$(CAT)_6$	155	56	TAMRA	MK238361
	R: GTGCAGTGTTCTGTCTCACG					
PasE205	F: CTCCCGTACCTTCGATCACG	$(CGC)_5$	135	52	ROX	MK238363
	R: TCTTCGGATGGAGGGTCACT					
PasE208	F: CAGTGTGAGCTCAACGAGGT	$(CGG)_6$	173	56	FAM	MK238364
	R: TCCTCGGCACTCCCTTAGAT					
PasE268	F: TTGATTTCACTCCCGGCGAA	$(GA)_7$	163	56	FAM	MK238366
	R: ACTTTCTTGCCAGAGCGTGT					
PasE290	F: GCGAAAGATGAAGCGAAGAGG	$(GAA)_5$	160	53	TAMRA	MK238367
	R: TCCACCATGAAACTGAGGCT					
PasE304	F: TCCATGTAACAAGTAAGCGGCTA	$(GAT)_7$	114	56	ROX	MK238369
	R: TCGTGTCTTCTCATTACTCCACA					

PasE348	F: GCCGCCGATTCAAGAGATTC<br>R: ACGATTCACCTCCGAACCTC	$(TA)_6$	190	49	ROX	MK238370
PasE425	F: AACCCACCATCACCACCATC<br>R: GCTCGTCTTGAAACCGCATC	$(TC)_7$	157	53	ROX	MK238373
PasE447	F: GGGTGAGGTGGAGTTAAGGC<br>R: CTTCCGGTATTGCACCCACA	$(TCG)_7$	156	52	FAM	MK238374
PasE452	F: GTGGTTGTGGAAAGAGAGGGT<br>R: GTCTGCTGCTGATGCTGTTG	$(TCT)_5$	178	56	HEX	MK238375

Note: F represents the forward primer, R means the reverse primer.

[*] represents the fluorescent dye should be concatenated in the 5’-end of the forward primer.
