# Supplementary material for: Phylogeography and Population Demography of Parrotia subaequalis, a Hamamelidaceous Tertiary Relict ‘Living Fossil’ Tree Endemic to East Asia Refugia: Implications from Molecular Data and Ecological Niche Modeling
Source: Plants (Basel). 2025 Jun 7;14(12):1754. doi: 10.3390/plants14121754 (PMC12197062; doi:10.3390/plants14121754)
Supplement: Supplementary file 1 [file plants-14-01754-s001.zip › Table S4.pdf]

Table S4. 19 bioclimatic variables for ENM analysis.

Code	Bioclimatic variable	Percent contribution
BIO1	Annual Mean Temperature	–
BIO2	Mean Diurnal Range (Mean of monthly (max temp-min temp))	–
BIO3*	Isothermality (BIO2/BIO7) (* 100)	0.2%
BIO4*	Temperature Seasonality (standard deviation *100)	19.1%
BIO5*	Max Temperature of Warmest Month	34.4%
BIO6	Min Temperature of Coldest Month	–
BIO7	Temperature Annual Range (BIO5-BIO6)	–
BIO8*	Mean Temperature of Wettest Quarter	10.1%
BIO9*	Mean Temperature of Driest Quarter	0.2%
BIO10	Mean Temperature of Warmest Quarter	–
BIO11*	Mean Temperature of Coldest Quarter	4.1%
BIO12*	Annual Precipitation	0.4%
BIO13*	Precipitation of Wettest Month	0.1%
BIO14	Precipitation of Driest Month	–
BIO15*	Precipitation Seasonality (Coefficient of Variation)	2.2%
BIO16	Precipitation of Wettest Quarter	–
BIO17*	Precipitation of Driest Quarter	24.8%
BIO18*	Precipitation of Warmest Quarter	4.5%
BIO19	Precipitation of Coldest Quarter	–

Note: The 11 selected bioclimatic variables in this study are marked by *.

"–" means no value.
