# Supplementary material for: Phylogeography and Population Demography of Parrotia subaequalis, a Hamamelidaceous Tertiary Relict ‘Living Fossil’ Tree Endemic to East Asia Refugia: Implications from Molecular Data and Ecological Niche Modeling
Source: Plants (Basel). 2025 Jun 7;14(12):1754. doi: 10.3390/plants14121754 (PMC12197062; doi:10.3390/plants14121754)
Supplement: Supplementary file 1 [file plants-14-01754-s001.zip › Table S5.pdf]

Table S5. Chloroplast DNA sequence polymorphisms detected in *Parrotia subaequalis* at three intergenic spacer (*psbC–psbZ*, *accD–psaI*, *ndhD–psaC*) regions, identifying 13 chlorotypes (H1–H13).

Chlorotype	Nucleotide position										
	psbC–psbZ			*accD–psaI*				*ndhD–psaC*			
	26	117	142	432	542	575	596	914	1170	1330	1467
H1	A	G	G	C	1[a]	T	A	G	C	A	A
H2	G	·	A	T	·	·	C	·	·	T	·
H3	G	·	A	T	·	·	C	A	·	T	G
H4	G	·	A	T	·	·	C	·	·	T	G
H5	G	·	A	·	·	·	·	A	·	T	G
H6	G	·	·	T	·	·	C	·	·	T	·
H7	G	·	A	·	0	·	·	·	·	·	·
H8	G	·	A	T	·	·	C	·	A	T	·
H9	·	·	A	·	·	·	·	·	·	·	·
H10	G	·	A	T	·	·	C	·	·	·	·
H11	G	·	A	·	·	·	·	·	·	·	·
H12	G	A	A	T	·	·	C	·	·	T	·
H13	G	·	A	T	·	G	C	·	·	T	·

Note: All sequences were compared to the reference chlorotype H1. Numbers '1/0' in sequences represented presence/absence of length polymorphisms. [a] represented ATACGA.
