# Supplementary material for: Phylogeography and Population Demography of Parrotia subaequalis, a Hamamelidaceous Tertiary Relict ‘Living Fossil’ Tree Endemic to East Asia Refugia: Implications from Molecular Data and Ecological Niche Modeling
Source: Plants (Basel). 2025 Jun 7;14(12):1754. doi: 10.3390/plants14121754 (PMC12197062; doi:10.3390/plants14121754)
Supplement: Supplementary file 1 [file plants-14-01754-s001.zip › Table S6.pdf]

Table S6. Genetic characteristics of cpDNA for 21 *Parrotia subaequalis* populations.

Popuation code	Chlorotype distribution	*h*	π ($\times 10^{-3}$)
SYC	H2, H8–10	0.370	0.880
TXC	H11	0.000	0.000
SLG	H2	0.000	0.000
DLX	H6	0.000	0.000
SJD	H6	0.000	0.000
GDS	H2	0.000	0.000
LWS	H2, H7	0.309	0.820
QSW	H2	0.000	0.000
ZXC	H13	0.000	0.000
SJW	H2	0.000	0.000
LHJ	H2	0.000	0.000
QL	H2	0.000	0.000
WFS	H12	0.000	0.000
YSH	H2, H4	0.441	0.290
TTS	H3	0.000	0.000
TJZ	H3, H5	0.118	0.160
TX	H3	0.000	0.000
DXG	H2	0.000	0.000
JCY	H2	0.000	0.000
HNZ	H2-4	0.604	0.710
WLS	H1	0.000	0.000
Mean value	–	0.088	0.136
Total value	–	0.737	1.210

Note: "–" represents no value.
