# Supplementary material for: Phylogeography and Population Demography of Parrotia subaequalis, a Hamamelidaceous Tertiary Relict ‘Living Fossil’ Tree Endemic to East Asia Refugia: Implications from Molecular Data and Ecological Niche Modeling
Source: Plants (Basel). 2025 Jun 7;14(12):1754. doi: 10.3390/plants14121754 (PMC12197062; doi:10.3390/plants14121754)
Supplement: Supplementary file 1 [file plants-14-01754-s001.zip › Table S7.pdf]

Table S7. Analysis of molecular variance (AMOVA) of cpDNA sequences (*psbC–psbZ*, *accD–psaI*, *ndhD–psaC*) data of the populations of *Parrotia subaequalis*.

Source of variation	d.f.	Sum of squares	Variance components	Percentage of variation (%)
Among populations	20	269.850	0.83242 Va	87.62
Within populations	318	37.389	0.11758 Vb	12.38
Total	338	307.239	0.94999	
