# Supplementary material for: Phylogeography and Population Demography of Parrotia subaequalis, a Hamamelidaceous Tertiary Relict ‘Living Fossil’ Tree Endemic to East Asia Refugia: Implications from Molecular Data and Ecological Niche Modeling
Source: Plants (Basel). 2025 Jun 7;14(12):1754. doi: 10.3390/plants14121754 (PMC12197062; doi:10.3390/plants14121754)
Supplement: Supplementary file 1 [file plants-14-01754-s001.zip › Table S8.pdf]

Table S8. Summary statistics of the genetic diversity of the 16 polymorphic EST-SSR loci.

Locus	N_A	Ho	H_E	H_T	PIC	F_{ST}	F_{IS}
PasE6	4	0.420	0.532	0.530	0.436	0.252	-0.044
PasE27	6	0.624	0.501	0.512	0.418	0.100	-0.386
PasE83	5	0.566	0.563	0.567	0.493	0.130	-0.160
PasE108	8	0.378	0.413	0.401	0.391	0.117	-0.029
PasE156	5	0.434	0.496	0.501	0.454	0.147	-0.030
PasE178	5	0.480	0.582	0.581	0.492	0.199	-0.028
PasE188	6	0.476	0.502	0.483	0.451	0.118	-0.047
PasE205	3	0.041	0.059	0.062	0.058	0.325	-0.016
PasE208	4	0.488	0.555	0.554	0.506	0.200	-0.114
PasE268	5	0.537	0.520	0.520	0.406	0.112	-0.160
PasE290	5	0.249	0.249	0.245	0.237	0.138	-0.148
PasE304	2	0.427	0.441	0.442	0.344	0.157	-0.146
PasE348	5	0.434	0.408	0.434	0.374	0.174	-0.226
PasE425	4	0.449	0.465	0.461	0.361	0.133	-0.136
PasE447	5	0.285	0.284	0.294	0.265	0.180	-0.231
PasE452	2	0.227	0.285	0.268	0.244	0.218	-0.003
Mean	4.625	0.407	0.428	0.428	0.371	0.169	-0.119

Note: N_A, Number of alleles per locus; Ho, Observed heterozygosity; H_E, Expected heterozygosity; H_T, Total genetic diversity; PIC, Polymorphic information content; F_{ST}, Genetic differentiation index; F_{IS}, Inbreeding coefficient.
