# Supplementary material for: Phylogeography and Population Demography of Parrotia subaequalis, a Hamamelidaceous Tertiary Relict ‘Living Fossil’ Tree Endemic to East Asia Refugia: Implications from Molecular Data and Ecological Niche Modeling
Source: Plants (Basel). 2025 Jun 7;14(12):1754. doi: 10.3390/plants14121754 (PMC12197062; doi:10.3390/plants14121754)
Supplement: Supplementary file 1 [file plants-14-01754-s001.zip › Table S9.pdf]

Table S9. Summary statistics of the genetic diversity of the 21 populations of *Parrotia subaequalis* based on the 16 polymorphic EST-SSR loci.

Population code	N_A	H_O	H_E	H_T	R_S	F_{IS}
SYC	42	0.406	0.350	0.357	2.218	-0.138
TXC	31	0.448	0.312	0.329	1.938	-0.361
SLG	38	0.395	0.386	0.399	2.207	0.011
DLX	39	0.384	0.334	0.341	2.049	-0.126
SJD	37	0.378	0.319	0.325	1.999	-0.162
GDS	34	0.384	0.311	0.317	1.885	-0.213
LWS	50	0.464	0.419	0.426	2.453	-0.088
QSW	35	0.375	0.328	0.335	1.992	-0.118
ZXC	36	0.448	0.377	0.386	2.062	-0.160
SJW	42	0.382	0.339	0.348	2.136	-0.098
LHJ	37	0.472	0.400	0.409	2.139	-0.154
QL	41	0.500	0.409	0.417	2.242	-0.200
WFS	38	0.300	0.316	0.325	2.028	0.076
YSH	47	0.381	0.395	0.406	2.426	0.061
TTS	40	0.419	0.398	0.408	2.249	-0.025
TJZ	43	0.478	0.373	0.380	2.129	-0.259
TX	36	0.281	0.296	0.304	1.957	0.075
DXG	44	0.364	0.385	0.398	2.313	0.086
JCY	41	0.447	0.384	0.390	2.120	-0.146
HNZ	50	0.355	0.394	0.409	2.439	0.130
WLS	39	0.457	0.332	0.337	2.096	-0.357
Mean	40	0.406	0.360	0.369	2.146	-0.103

Note: N_A, Number of alleles per population; H_O, Observed heterozygosity; H_E, Expected heterozygosity; H_T, Total genetic diversity; R_S, Allelic richness; F_{IS}, Inbreeding coefficient.
