# Supplementary figures and images for: Phylogeography and Population Demography of Parrotia subaequalis, a Hamamelidaceous Tertiary Relict ‘Living Fossil’ Tree Endemic to East Asia Refugia: Implications from Molecular Data and Ecological Niche Modeling
Source: Plants (Basel). 2025 Jun 7;14(12):1754. doi: 10.3390/plants14121754 (PMC12197062; doi:10.3390/plants14121754)

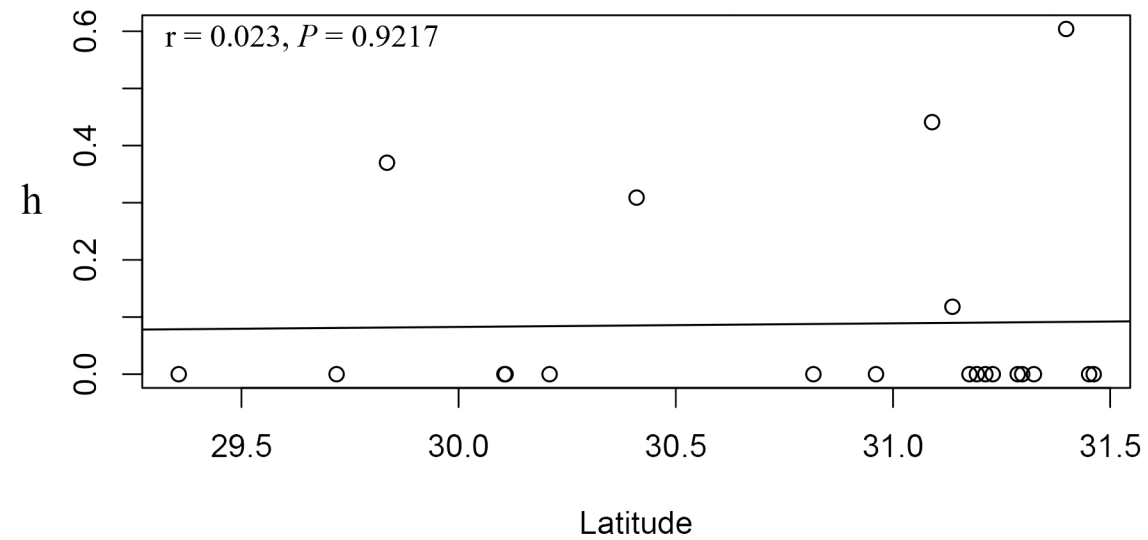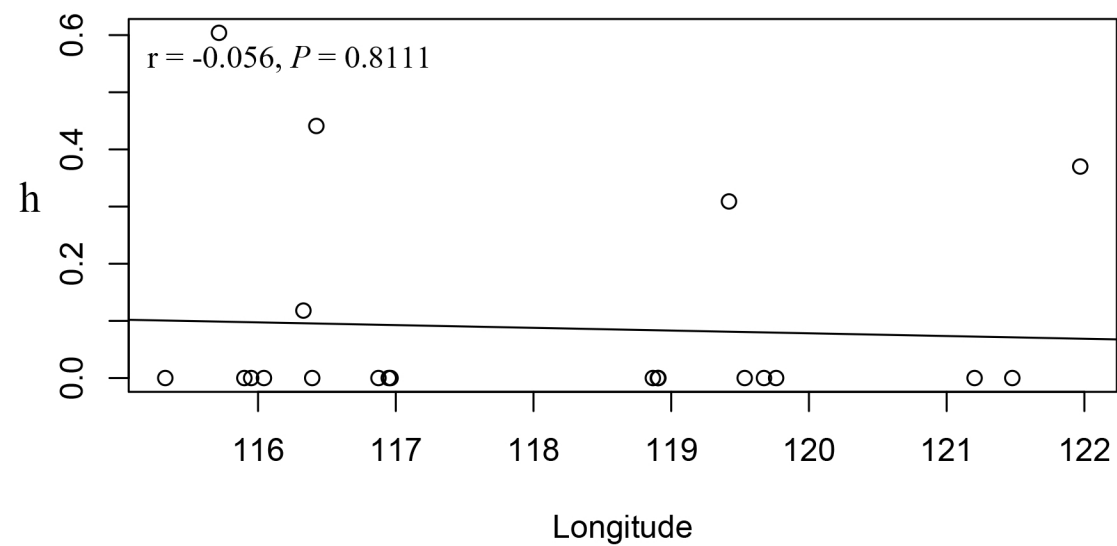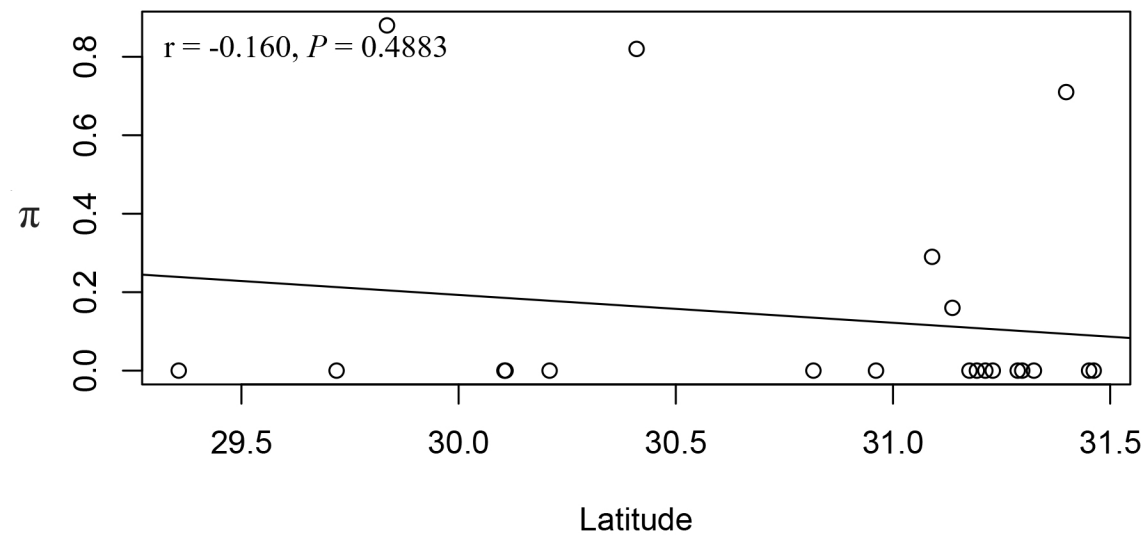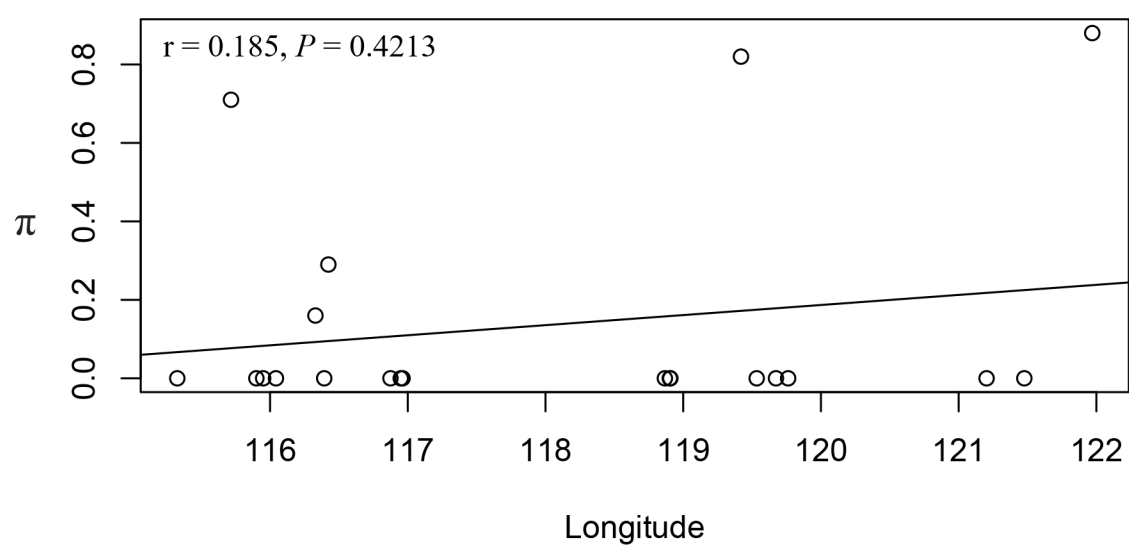

Supplement: Supplementary file 1 [file plants-14-01754-s001.zip › Figure S1.pdf]

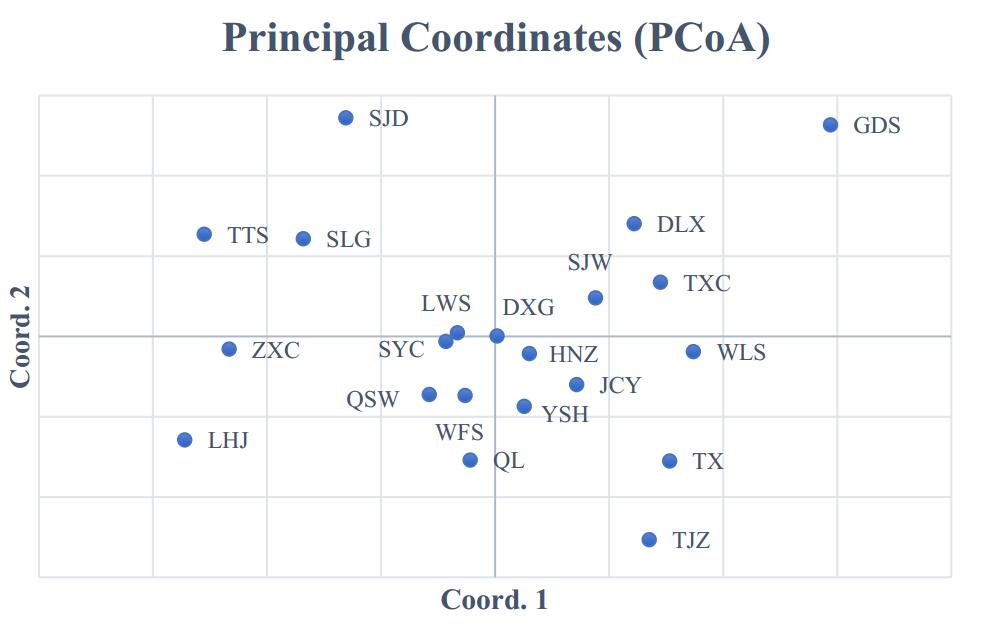

Supplement: Supplementary file 1 [file plants-14-01754-s001.zip › Figure S2.jpg]

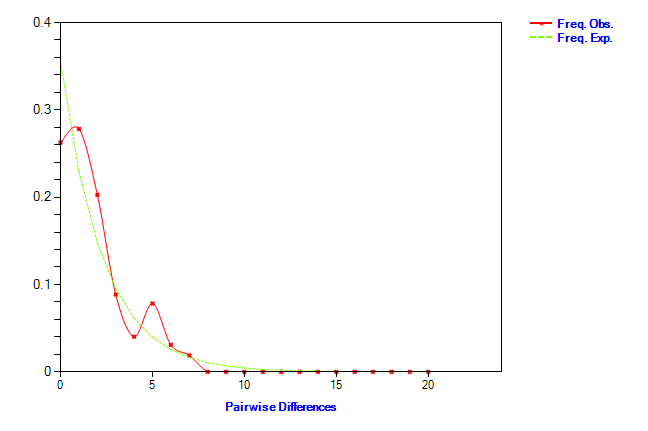

Supplement: Supplementary file 1 [file plants-14-01754-s001.zip › Figure S3.tif]

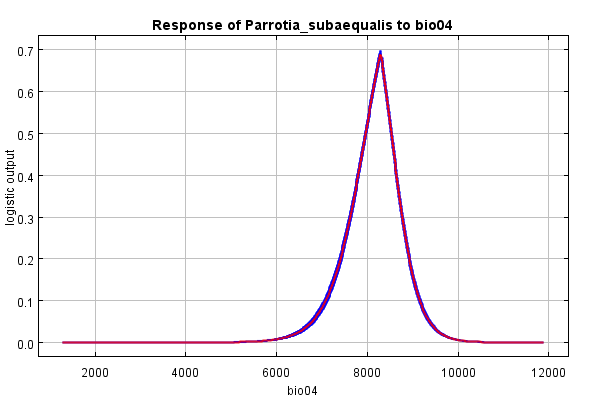

Supplement: Supplementary file 1 [file plants-14-01754-s001.zip › Figure S4.png]

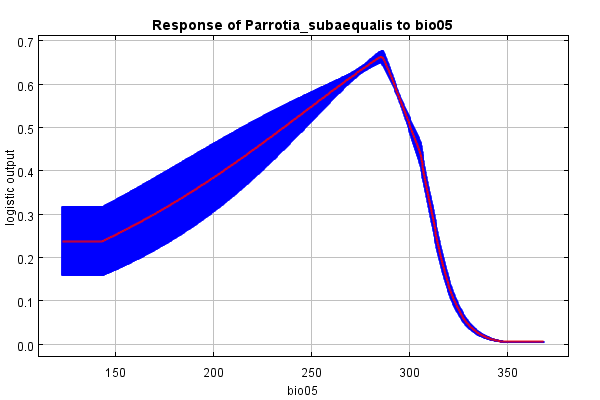

Supplement: Supplementary file 1 [file plants-14-01754-s001.zip › Figure S5.png]

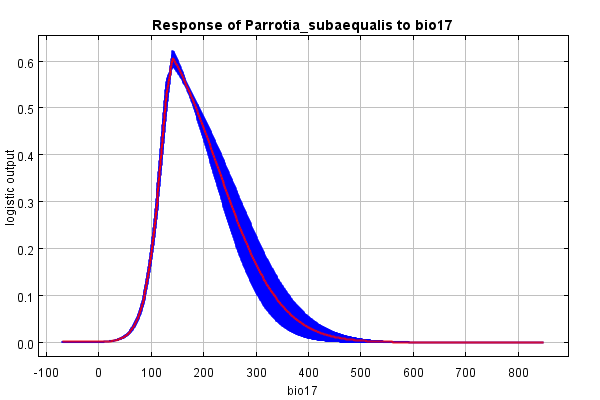

Supplement: Supplementary file 1 [file plants-14-01754-s001.zip › Figure S6.png]

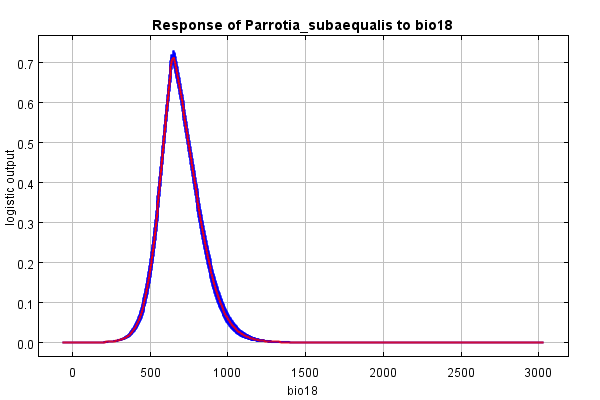

Supplement: Supplementary file 1 [file plants-14-01754-s001.zip › Figure S7.png]
